# RNAi Induces Innate Immunity through Multiple Cellular Signaling Pathways

**DOI:** 10.1371/journal.pone.0064708

**Published:** 2013-05-20

**Authors:** Zhongji Meng, Xiaoyong Zhang, Jun Wu, Rongjuan Pei, Yang Xu, Dongliang Yang, Michael Roggendorf, Mengji Lu

**Affiliations:** 1 Institute of Virology, University Hospital of Essen, University Duisburg-Essen, Essen, Germany; 2 Department of Infectious Diseases, Taihe Hospital, Hubei University of Medicine, Shiyan, China; 3 Department of Infectious Diseases, Union Hospital, Tongji Medical College, Huazhong University of Science and Technology, Wuhan, China; 4 Department of Microbiology, Tongji Medical College, Huazhong University of Science and Technology, Wuhan, China; McMaster University, Canada

## Abstract

**Background & Aims:**

Our previous results showed that the knockdown of woodchuck hepatitis virus (WHV) by RNA interference (RNAi) led to upregulation of interferon stimulated genes (ISGs) in primary hepatocytes. In the present study, we tested the hypothesis that the cellular signaling pathways recognizing RNA molecules may be involved the ISG stimulation by RNAi.

**Methods:**

Primary murine hepatocytes (PMHs) from wild type mice and WHV transgenic (Tg) mice were prepared and treated with defined siRNAs. The mRNA levels of target genes and ISGs were detected by real-time RT-PCR. The involvement of the signaling pathways including RIG-I/MDA5, PKR, and TLR3/7/8/9 was examined by specific inhibition and the analysis of their activation by Western blotting.

**Results:**

In PMHs from WHV Tg mice, specific siRNAs targeting WHV, mouse β-actin, and GAPDH reduced the levels of targeted mRNAs and increased the mRNA expression of IFN-β, MxA, and IP-10. The enhanced ISG expression by siRNA transfection were abolished by siRNA-specific 2′-O-methyl antisense RNA and the inhibitors 2-AP and chloroquine blocking PKR and other TLR-mediated signaling pathways. Furthermore, Western blotting revealed that RNAi results in an increase in PKR phosphorylation and nuclear translocation of IRF3 and NF-êB, indicating the possible role of IRF3 in the RNAi-directed induction of ISGs. In contrast, silencing of RIG-I and MDA5 failed to block RNAi-mediated MxA induction.

**Conclusions:**

RNAi is capable of enhancing innate immune responses through the PKR- and TLR-dependent signaling pathways in primary hepatocytes. The immune stimulation by RNAi may contribute to the antiviral activity of siRNAs in vivo.

## Introduction

RNA interference (RNAi) is a natural process whereby double-stranded RNA (dsRNA) induces sequence-specific degradation of homologous messenger RNA (mRNA). This process is mediated by small interfering RNAs (siRNAs) with a length of 21 to 23 nucleotides [1]. RNAi is a revolutionary approach for basic biological research as well as drug development. The ability to manipulate mammalian cells with RNAi may provide critical insights into the mechanisms underlying human disease and accelerate the development of treatments for cancer, infectious diseases, and various other disorders. The RNAi approach has been widely used for drug development, and several phase I and II clinical trials are in progress [2]–[4]. RNAi also provides a promising approach for the specific treatment of HBV infection. Various recent studies have demonstrated the effectiveness of specific siRNAs for inhibiting HBV gene expression and viral replication [5]–[14].

In our attempt to inhibit the expression of woodchuck hepatitis virus (WHV) in primary woodchuck hepatocytes (PWHs) naturally infected with WHV, we found that RNAi-mediated suppression of WHV enhanced the expression of cellular genes such as MxA and MHC-I, which suggests that specific siRNAs are able to inhibit hepadnavirus replication and enhance the expression of cellular genes relevant for antiviral action [14]. The mechanism underlying this enhanced expression of cellular antiviral genes requires further investigation. It has been reported that the cleavage of cellular RNAs by RNase L produces small RNAs that are able to activate IFN-β [15]. Therefore, the production of small RNA fragments and triggering of IFN-β expression by siRNA-directed RNA degradation should also be investigated. The lack of woodchuck-specific reagents prevents this process from being examined in woodchuck cells. It is noteworthy, however, that enhanced expression of MxA and MHC-I by siRNA treatment does not occur in established human hepatoma cell lines that contain replicating HBV (Meng and Lu, unpublished results), possibly because of defective IFN-β signaling pathways in these hepatoma cells [16].

Our previous results in PWHs isolated from woodchucks chronically infected with WHV showed that specific siRNA inhibition of WHV replication and downregulation of WHV transcripts upregulated interferon-stimulated genes (ISGs) and inflammatory cytokines. Because HBV may counteract host antiviral effector mechanisms by directly inhibiting the IFN-β signaling pathway [17], downregulation of the IFN-β-inducible MxA promoter through direct interaction with precore/core proteins or the inhibition of proteasomal activities may occur in an HBX-dependent manner [18], [19]. Thus, it is reasonable that RNAi of WHV may reduce the amount of WHV protein and thereby facilitate cellular gene expression, i.e., prevent antigen tolerance in the host innate immune system, particularly the IFN-β signaling pathway [20], [21].

Recently, we constructed a WHV transgenic (Tg) mouse model in which we showed high levels of WHV transcripts and DNA replicative intermediates in the liver (Meng et al., submitted). In primary hepatocytes (PMHs) from the WHV Tg mouse model, we verified that RNAi-mediated suppression of WHV enhances the expression of cellular genes. Moreover, RNAi-mediated suppression of cellular housekeeping genes such as GAPDH and β-actin also resulted in enhanced cellular gene expression. To determine the mechanism underlying the RNAi-mediated induction of IFN-β, the signaling pathways involved in recognition of small RNA molecules and IFN-β induction were investigated in PMHs and PWHs.

## Materials and Methods

### siRNA and inhibitors

The WHV-specific siRNA siWHx and the control siRNA (siGFP) were described previously [14]. The mouse-specific siRNAs siActin, siGAPDH, siGAPDH-1, siGAPDH-2, si-28S, and the siWHx-specific inhibitor (2′-O-methyl antisense RNA) were purchased from Qiagen (Düsseldorf, Germany). The target sequences of the siRNAs were listed in Table 1. All the siRNAs were unmodified. There is a 2 ‘T’ overhang at the 3′ end of the sense strand of the siRNAs. siGAPDH was usually used for the experiments, as it is the most effective siRNA targeting mouse GAPDH mRNA among the tested siRNAs. The PKR inhibitor 2-aminopurine (2-AP) and an inhibitor of endosomal acidification (chloroquine) were purchased from Invivogen (San Diego, CA). The PKR inhibitor (PKR-I) was provided by Merck (Darmstadt, Germany).

**Table 1:** siRNA target sequences.

siRNA	Target sequence	Target gene
siWHx	5′-AAAGATCAATTATTAACTAAA-3′	WHV
siGFP	5′-CGGCAAGCTGACCCTGAAGTTCAT-3′	GFP
siActin	5′-CACTGACTTGAGACCAATAAA-3′	m-Actinb
siGAPDH-1	5′-CAGCTCGTCCCGTAGACAAA-3′	m-GAPDH
siGAPDH-2	5′-CACGGCAAATTCAACGGCACA-3′	m-GAPDH
siGAPDH	5′-AAGGTCGGTGTGAACGGATTT-3′	m-GAPDH
si-28S	5′-AACGGTAACGCAGGTGTCCTA-3′	m-28S rRNA

### Woodchucks and WHV Tg mice

Woodchucks were purchased from North Eastern Wildlife (Ithaca, NY, USA). WHV Tg mice were constructed and bred in our institute (Meng et al., submitted). All animals were kept in the laboratory animal center of the University of Essen. The experiments were conducted in accordance with the Guide for the Care and Use of Laboratory Animals and were reviewed and approved by the local Animal Care and Use Committee of the district government (Düsseldorf, Germany). For the preparation of primary hepatocytes, no specific permission was needed, as the experiments were not carried out with living animals.

### Culture of primary hepatocytes and siRNA transfection

Primary hepatocytes from WHV-infected woodchucks and WHV Tg mice were prepared by perfusion of the liver with collagenase IV (Sigma, Munich, Germany) as described previously [14], [22]. The viability of prepared hepatocytes was assessed by trypan blue dye exclusion and exceeded 80% in all experiments. The cells were plated at a density of 1.5×10^6^ cells per well on collagen I-coated 6-well plates in Williams medium (Gibco, Karlsruhe, Germany) supplemented with 1 mg/ml hydrocortisone (Sigma), 10 µg/ml insulin (Serva, Amsterdam, The Netherlands), 50 µg/ml gentamicin (Sigma), 50 IU/ml penicillin-streptomycin (Gibco), 2 mM glutamine (Gibco), 1.25 mg/ml inosine (Serva), and 1% DMSO (Merck, Darmstadt Germany).

Transfection of primary hepatocytes with siRNAs was performed on day 2 of in vitro culture except where otherwise specified. Lipofectamine 2000 (Invitrogen, Karlsruhe, Germany) was used according to the manufacturer's instructions. In each well, 150 pmol siRNA and 5 µl of Lipofectamine 2000 were applied in a final volume of 1.5 ml of Opti-MEM. After 5 h, the medium was replaced with fresh culture medium.

### RNA preparation and real-time reverse transcriptase (RT)-PCR

Total RNA was purified using the RNeasy Mini Kit (Qiagen) according to the manufacturer's instructions. One-step real-time RT-PCR was performed for 100 ng of total RNA using the QuantiFast SYBR Green RT-PCR Kit (Qiagen) on a Light Cycler^TM^ instrument (Roche) with the following program: 50°C for 10 min for reverse transcription followed by 45 cycles of PCR (95°C for 10 sec and 60°C for 30 sec). The primers used for the amplification of WHV, woodchuck actin, IFN-β, MxA, and IP-10, are listed in Table 2. The primers for mouse actin, GAPDH, IFN-β, MxA, and IP-10, were purchased from Qiagen. The relative levels of mRNA copies were determined using a standard curve constructed from the corresponding cDNA fragments. The copy numbers of WHV, woodchuck and mouse IFN-β, MxA, and IP-10 transcripts were normalized against those of woodchuck or mouse β-actin transcripts, respectively, and presented as copies per 1000 copies of β-actin.

**Table 2:** Primer sequences for the amplification of WHV and woodchuck genes.

Amplicon	Polarity	Nucleotide sequence (5′ → 3′)	Position	Reference
WHV	Sense	TGGGGCATGGACATAGATCCCTA	2015	M19183
	Antisense	AAGATCTCTAAATGACTGTATGTTCCG	2467	M19183
wβ-actin	Sense	TGGAATCCTGTGGCATCCATGAAAC	1	AY170121
	Antisense	TAAAACGCAGCTCAGTAACAGTCCG	346	AY170121
wMxA	Sense	GGAGGGAGGAGAAGAGGAAA	66	EU503128
	Antisense	CTGGAGATGCGGTTGTGAG	189	EU503128
wIP-10	Sense	ACATCTCTTCTCCCCGCTCT	121	EU564728
	Antisense	TCCTGCAAGTGAATCTTGTCC	190	EU564728
wIFN-β	Sense	TCTCCACCACAGCTCTTTCC	39	DQ402072
	Antisense	GGCCTTTCATTCAACTGCTCT	141	DQ402072

The underlined portion is the specific cleavage sites of restriction enzyme *BglII*.

### Preparation of cell lysates and Western blotting analysis

Cultured hepatocytes were washed once with ice-cold phosphate-buffered saline (PBS) and lysed in 1× SDS sample buffer (62.5 mM Tris-HCl, pH 6.8, 2% w/v SDS, 10% glycerol, 50 mM DTT, 0.01% w/v bromophenol blue) supplemented with a protease inhibitor cocktail (Roche) and a phosphatase inhibitor cocktail (Sigma-Aldrich). Protein samples were subjected to SDS-PAGE and Western blotting with primary antibodies that selectively recognized mouse RIG-I, IPS-1, and phosphorylated forms of PKR (Cell Signaling Technology, Danvers, MA). To determine the amounts of loaded protein, the blots were stripped and reprobed with an anti-â-actin (Sigma-Aldrich) antibody. To detect the nuclear translocation of the NF-êB p65 subunit and IRF3, cytoplasmic and nuclear extracts were prepared using NE-PER nuclear and cytoplasmic protein extraction reagents (Pierce, Rockford, IL). Protein concentrations were determined using a Bio-Rad protein assay kit (Bio-Rad Laboratories, Hercules, CA). The nuclear NF-êB p65 subunit and IRF3 were detected by Western blotting using specific antibodies (Santa Cruz). Protein bands were visualized using ECL Plus Western blotting detection reagents (GE Healthcare), followed by exposure to Kodak Bio-Max films (Carestream Health, Paris, France).

## Results

### Silencing of viral and host genes upregulates IFN-β and ISGs

**Figure 1:**
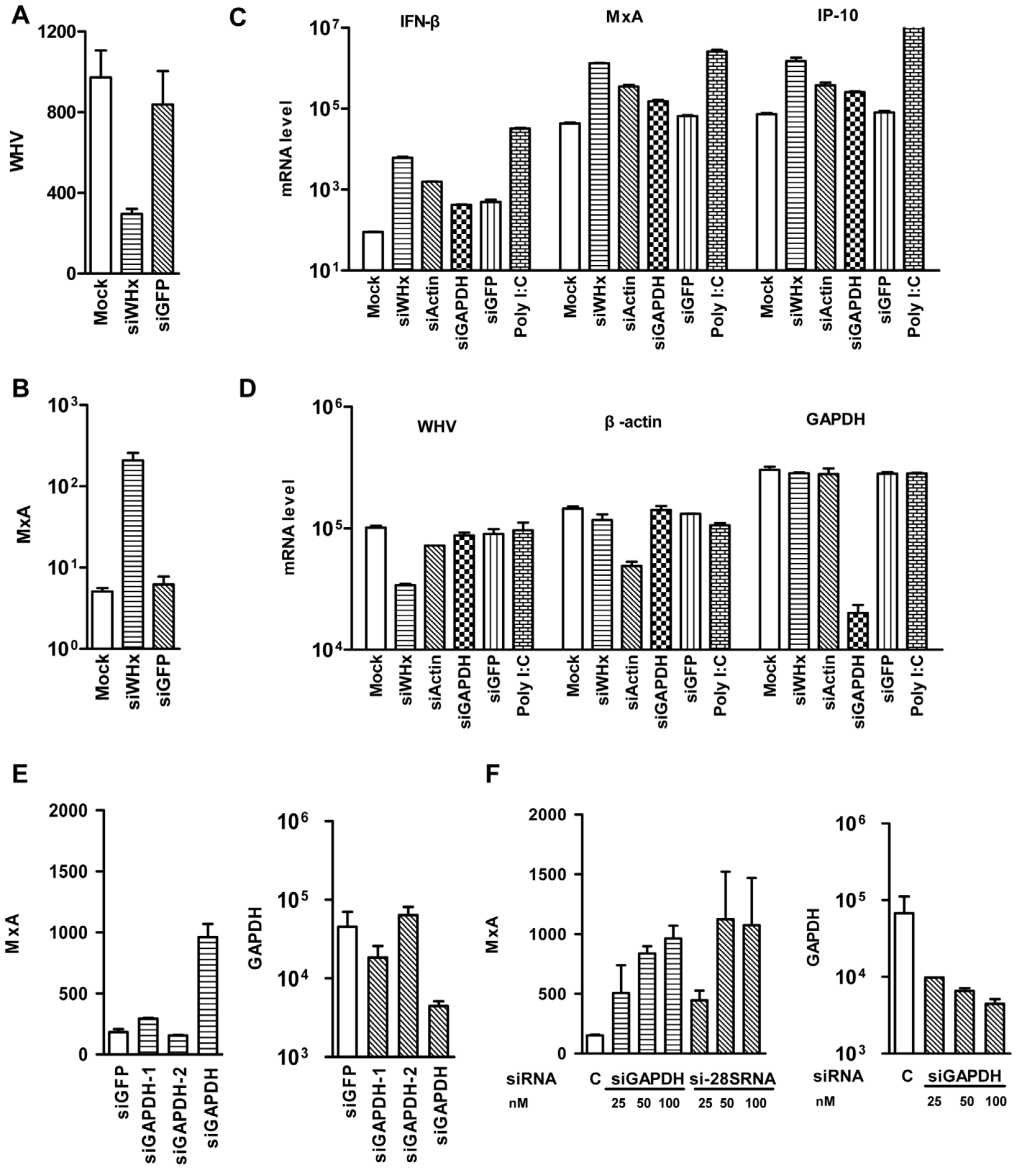
Interference targeted against WHV and housekeeping genes led to upregulation of IFN-β, MxA, and IP-10. A and B. PWHs isolated from woodchucks with chronic WHV infection were transfected with 100 nM siWHx or siGFP as a control, and the mRNA levels of WHV and woodchuck MxA were determined by real-time RT-PCR 48 h posttransfection. C and D. PMHs isolated from WHV Tg mice were transfected with 100 nM siWHx or the mouse-specific siRNAs siActin, siGAPDH, or siGFP and poly I:C as controls, and the mRNA levels of WHV, mouse actin, GAPDH, IFN-β, MxA, and IP-10 were determined by real-time RT-PCR 48 h posttransfection. E and F. PMHs isolated from C57BL/6 mice were transfected with siGAPDH, siGAPDH-1, siGAPDH-2, si-28S, or siGFP as a control, the mRNA levels of mouse MxA and GAPDH were determined by real-time RT-PCR 6 h post transfection.

Our previous results in PWHs showed that silencing of WHV gene expression and replication enhanced the expression of cellular antiviral proteins such as MxA and MHC-I (Fig. 1A and B) [14]. Here, siWHx was tested in PMHs isolated from WHV Tg mice. PMHs were transfected with siWHx, and the RNA levels of WHV, mouse β-actin, GAPDH, IFN-β, MxA, and IP-10 were determined by real-time RT-PCR. Knockdown of WHV mRNA significantly increased the mRNA levels of mouse IFN-β, MxA, and IP-10 in PMHs (Fig. 1C and D). We then determined whether interference targeted against housekeeping genes such as GAPDH and β-actin could provide similar results. PMHs were transfected with validated siRNAs targeting mouse GAPDH and β-actin. In both cases, the decrease in the mRNA levels of mouse GAPDH and β-actin was accompanied by an increase in the mRNA levels of mouse IFN-β, MxA, and IP-10. Furthermore, the other two siRNAs siGAPDH-1 and siGAPDH-2 targeting mouse GAPDH were tested. Compared with the siGAPDH, siGAPDH-1 was less effective and led to a lower level of MxA upregulation. siGAPDH-2 did not induce any MxA upregulation, consistent with the fact that it was almost ineffective (Fig. 1E). In addition, the knockdown efficiency of GAPDH mRNAs and MxA induction showed the same dose-dependence if siGAPDH was used in different concentrations. For verification, a siRNA targeting at mouse 28S rRNA tested to show a similar dose-dependent effect of RNA knockdown and MxA induction (Fig 1F). In contrast, siWHx treatment did not affect the mRNA levels of mouse MxA in PMHs isolated from normal C57BL/6 mice, whereas GAPDH- and β-actin-specific siRNA treatment upregulated mouse MxA in a manner similar to that observed in PMHs isolated from WHV Tg mice (Fig. S1).

### Inhibition of RNAi blocks RNAi-directed MxA induction

**Figure 2:**
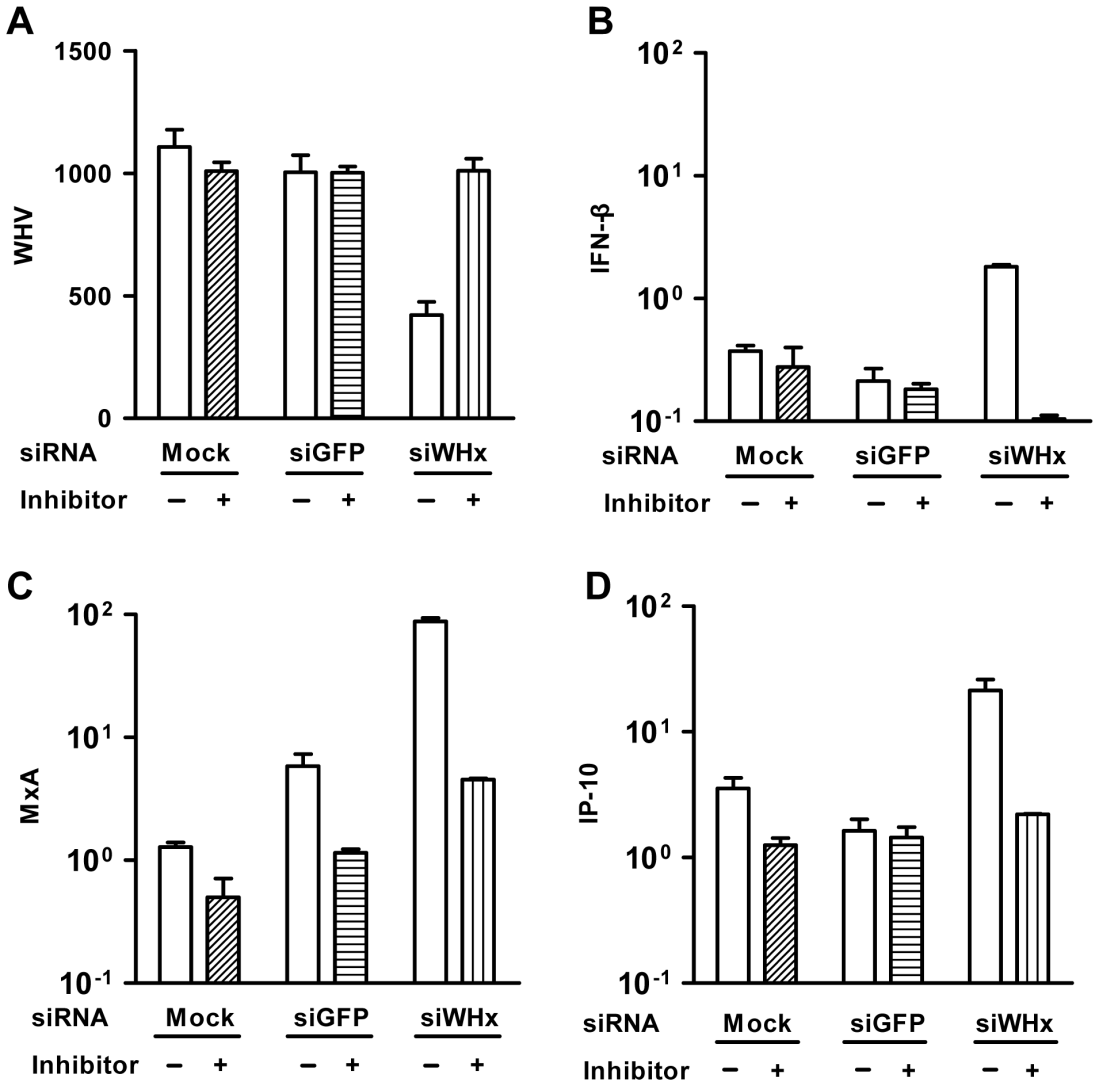
Inhibition of siRNA blocked the siWHx-mediated induction of IFN-β, MxA, and IP-10. PMHs isolated from WHV Tg mice were transfected with 100 nM of siWHx or cotransfected with 100 nM of siWHx and 100 nM of siWHx specific inhibitor (2′-O-methyl antisense RNA), and the mRNA levels of WHV and mouse IFN-β, MxA, and IP-10 were determined by real-time RT-PCR 48 h posttransfection.

The results described above suggest that RNAi or one or more factors produced during the RNAi process resulted in IFN-β/ISG induction. To test this hypothesis, the specific siRNA inhibitor 2′-O-methyl antisense RNA was used to block the RNAi process. WHV-positive PMHs were transfected with siWHx in the presence or absence of 2′-O-methyl antisense RNA, and the mRNA levels of WHV and mouse IFN-β, MxA, and IP-10 were determined by real-time RT-PCR 48 h posttransfection. In the absence of 2′-O-methyl antisense RNA, siWHx treatment resulted in a decrease in WHV mRNA and the significant upregulation of IFN-β, MxA, and IP-10. The presence of the siWHx inhibitor almost completely blocked the silencing effect of siWHx on WHV, and simultaneously, no obvious increase in the mRNA levels of IFN-β, MxA, and IP-10 was observed (Fig. 2).

### RNAi-mediated IFN-β/ISG induction showed a delayed kinetic compared with IFN induction by Poly I:C

**Figure 3:**
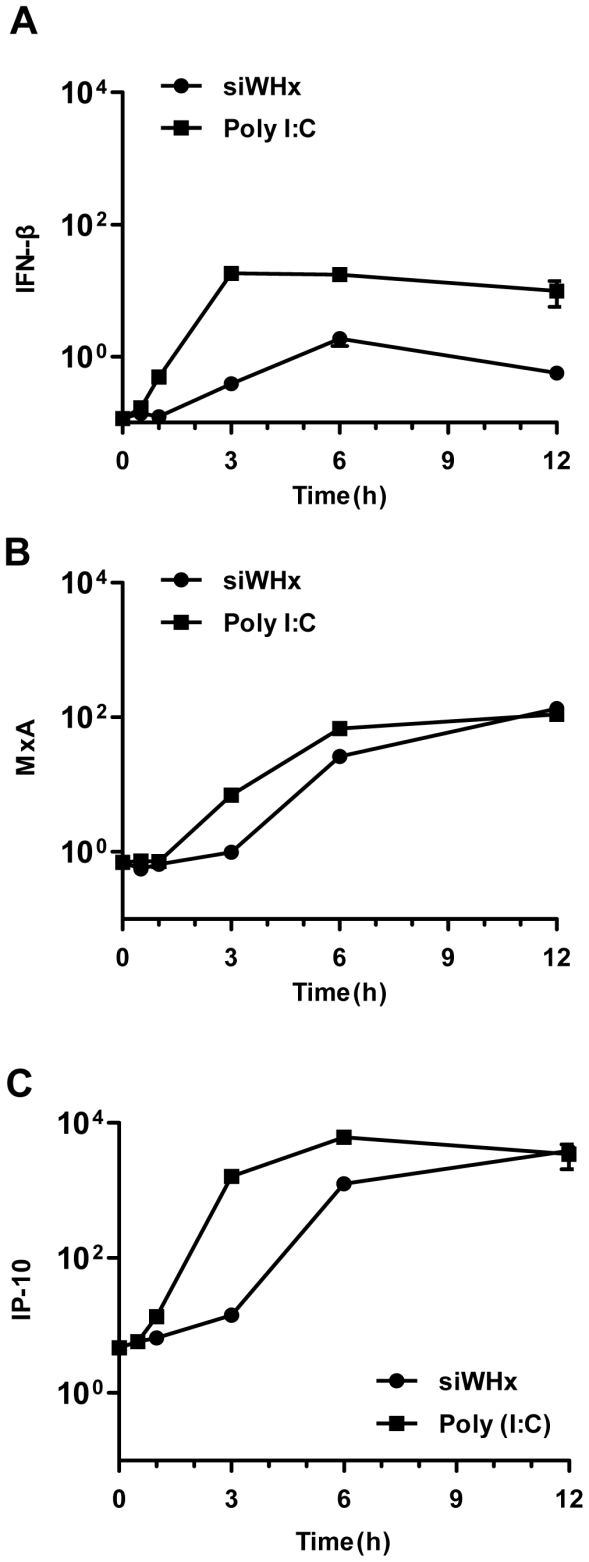
Comparison of RNAi-mediated induction of IFN-β/ISGs and poly I:C-stimulated induction of IFN-β/ISGs. PWHs isolated from woodchucks chronically infected with WHV were transfected with siWHx (100 nmol/L) or poly I:C (1 µg/mL), and the mRNA levels of woodchuck IFN-β (A), MxA (B), and IP-10 (C) were determined by real-time RT-PCR at 0, 0.5, 1, 3, 6, and 12 h posttransfection.

It has been reported that the cleavage of cellular RNA by RNase L produces small RNAs that are able to activate IFN-β [15]. Thus, RNAi-directed RNA degradation may also produce small RNA fragments and trigger the activation of the IFN-β gene. In the present study, the double-stranded small RNA fragment poly I:C, which is a strong inducer of IFN-β, was used as a positive control for IFN-β/ISG induction. PWHs isolated from woodchucks chronically infected with WHV were transfected with siWHx (100 nmol/L) or poly I:C (1 µg/mL). The mRNA levels of woodchuck IFN-β, MxA, and IP-10 increased at 1 h after transfection with poly I:C and at 3 to 6 h after transfection with siWHx (Fig. 3A, 3B, and 3C), which suggests that RNAi-mediated induction of IFN-β/ISGs may be a secondary effect of gene silencing. Based on the observation that RNAi-mediated IFN-β/ISG upregulation occurred later than poly I:C-mediated IFN-β/ISG upregulation, we consider that small RNA generated by RNAi may activate cellular signaling pathways that generate IFN-β responses. Furthermore, it takes hours for RNAi to cleave RNA and produce sufficient small RNAs to induce an IFN-β response. We therefore deduce that RNAi-mediated induction of IFN-β/ISGs is a definite secondary effect of gene silencing.

### Different cellular pathways are involved in RNAi-mediated MxA induction

Based on previous findings, at least 4 signaling pathways may recognize small RNA molecules and induce IFN-β production, including the RIG-I/MDA5 pathway, the TLR3 pathway, the TLR7/8 pathway, and the PKR pathway. It is reasonable that RNAi-generated small RNA cleavage products may activate one or more signaling pathways that induce IFN-β production.

**Figure 4:**
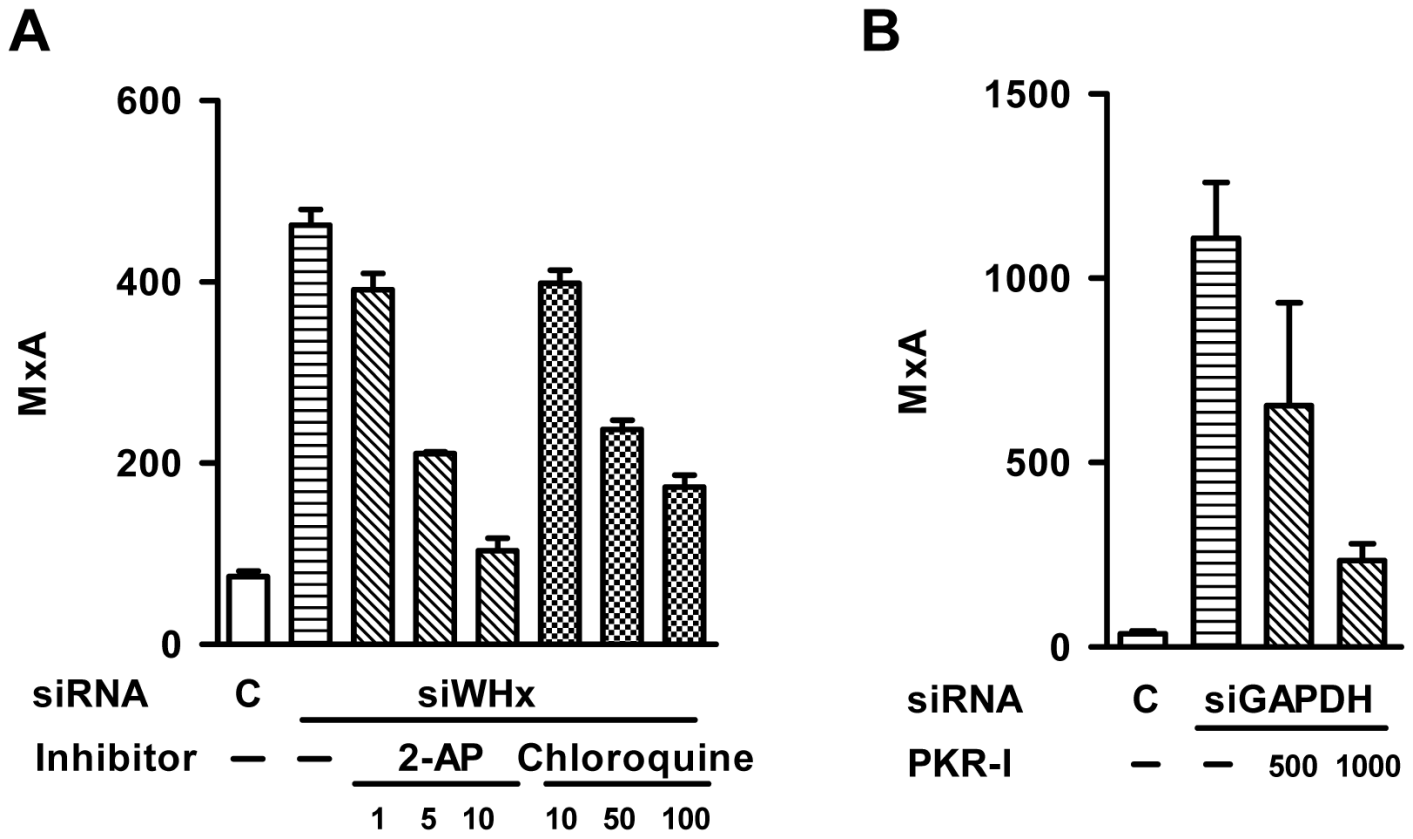
Inhibitors of PKR and TLR3/7/8 (chloroquine) blocked the RNAi-mediated induction of MxA. A. PMHs isolated from WHV Tg mice were pretreated with 2-AP (1, 5, 10 mM) or chloroquine (10, 50, 100 µM) followed by siWHx transfection, and the mRNA levels of mouse MxA were determined by real-time RT-PCR 6 h posttransfection. B. PMHs isolated from C57BL/6 mice were pretreated with PKR-I (0, 500, 1000 nM) for 4 h, followed by siWHx transfection, and the mRNA levels of mouse MxA were determined by real-time RT-PCR 6 h posttransfection. M, mock transfection; C, transfection with siGFP as a control.

In the present study, the involvement of PKR, TLR3, and TLR7/8 signaling pathways was tested using proper inhibitors. The drug 2-AP is a PKR inhibitor. Chloroquine is an inhibitor of endosomal acidification and known to inhibit TLR3, TLR7/8, and TLR9 mediated signaling. PMHs isolated from WHV Tg mice were pretreated with 2-AP or chloroquine and transfected with siWHx, and then mouse MxA mRNA levels were determined by real-time RT-PCR at 48 h posttransfection. In the absence of the inhibitors, siWHx treatment upregulated MxA by 3-fold, whereas pretreatment with 2-AP and chloroquine prevented the RNAi-mediated induction of MxA (Fig. 4A). Both inhibitors, especially the PKR inhibitor 2-AP, displayed a concentration-dependent inhibition of RNAi-mediated MxA upregulation (Fig. 4A). To further verify the role of PKR in the RNAi-mediated MxA upregulation, an alternative PKR inhibitor PKR-I was applied in PMHs transfected with siGAPDH. Consistently, PKR-I also inhibited the RNAi-mediated MxA upregulation by RNAi of GAPDH in a dose dependent manner (Fig. 4B). These results suggest that PKR and TLR3/7/8 pathways were involved in the induction of RNAi-mediated ISGs.

The RIG-I/MDA5 signaling pathway was tested using specific siRNAs that targeted mouse RIG-I/MDA5 and IPS1. PMHs isolated from WHV Tg mice were transfected with siWHx or cotransfected with the mouse-specific siRNAs siRIG-I, siMDA5, or siIPS1, and the mRNA levels of mouse MxA and protein levels of mouse RIG-I and IPS1 were determined at 48 h posttransfection by real-time RT-PCR and Western blotting, respectively. siWHx treatment upregulated MxA by more than 10-fold, whereas cotransfection with siRIG-I, siMDA5, and siIPS1 did not affect RNAi-mediated MxA induction (Fig. S2, upper panel). Western blotting demonstrated an RNAi-mediated decrease in the RIG-I protein level (Fig. S2, lower panel), suggesting that the RIG-I/MDA5 pathway was not involved in RNAi-mediated ISG induction.

### PKR is activated in the RNAi process

**Figure 5:**
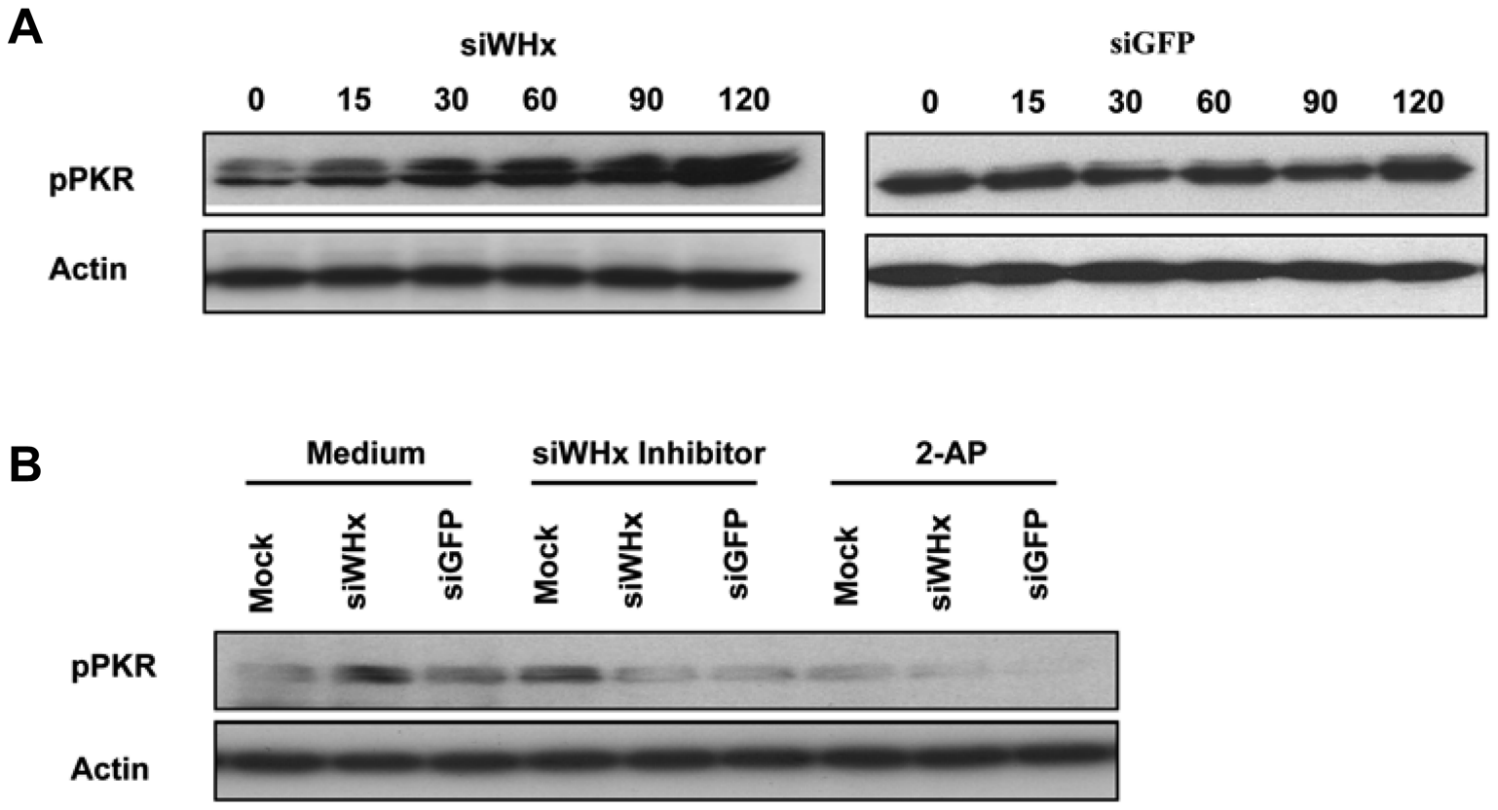
PKR phosphorylation in the RNAi process. **A.** PWHs isolated from woodchucks with chronic WHV infection were transfected with 100 nM siWHx or siGFP, and woodchuck actin and phosphorylated PKR were examined by Western blotting at 0, 15, 30, 60, 90, and 120 minutes posttransfection. B. PWHs isolated from woodchucks with chronic WHV infection were cotransfected with 100 nM siWHx inhibitor and siWHx or siGFP or pretreated with 2-AP and then transfected with 100 nM siWHx or siGFP. Woodchuck actin and phosphorylated PKR were examined by Western blotting at 120 minutes posttransfection.

The activation of PKR involves RNA-dependent autophosphorylation that results in the inhibition of translation. To test the activation of PKR in the RNAi process, we examined PKR phosphorylation following RNAi induction by siWHx treatment. PWHs isolated from woodchucks with chronic WHV infection were transfected with siWHx, and PKR phosphorylation was determined by Western blotting at 0, 15, 30, 60, 90, and 120 minutes posttransfection. As illustrated in Fig. 5A, siWHx treatment potently activated PKR phosphorylation, as expected. The phosphorylation of PKR was time-dependent with siWHx treatment: phosphorylation immediately increased at 15 min after siWHx treatment, peaked by 1 h, and remained increased until 2 h posttreatment. By contrast, treatment with control siRNA (si-GFP) that did not target specific RNA in PWHs did not affect PKR phosphorylation.

However, the presence of a siWHx-specific inhibitor that blocked siWHx-mediated RNA interference thoroughly compromised RNAi-mediated PKR phosphorylation (Fig. 5B). Similarly, the presence of the PKR inhibitor 2-AP blocked RNAi-directed PKR phosphorylation, as expected (Fig. 5B). Together, these results suggest that PKR was activated and that PKR phosphorylation increased in the RNAi process.

### RNAi results in the nuclear translocation of IRF3 and NF-κB

IRF3 and NF-κB are known to be important molecules downstream of the PKR, TLR-3, and TLR7/8 signaling pathways. Upon activation, IRF3 is phosphorylated, dimerized, and translocated from the cytoplasm into the nucleus. To investigate whether IRF3 and NF-κB are activated in the process of RNA interference, PWHs with WHV infection were treated with siWHx, and cytosolic and nuclear proteins were prepared at 0, 15, 30, 60, 90, and 120 minutes posttransfection. Cytosolic and nuclear IRF3 and NF-κB levels were examined by Western blotting. Within 30 min after siRNA treatment, cytosolic IRF3 and NF-κB levels decreased gradually, whereas nuclear IRF3 and NF-κB levels increased accordingly, which suggests that IRF3 and NF-κB were translocated from the cytoplasm into the nucleus. These results suggest that IRF3 and NF-κB were activated by the RNAi-mediated induction of ISGs.

## Discussion

The present study shows that RNAi targeted against WHV and the mouse housekeeping genes GAPDH and β-actin can upregulate IFN/ISGs. siWHx induced MxA upregulation only in PWHs with WHV transcripts, while siGAPDH and siActin were effective in PMHs from both WHV Tg and normal C57BL/6 mice, suggesting that MxA upregulation is a result of RNAi and independent on the presence of viral replication. We previously showed that RNAi with siWHx targeting WHV transcripts enhanced MxA expression to different levels, likely due to the abundance of mRNAs and the efficiency of RNAi [14]. In the present study, siRNAs (siGAPDH, siGAPDH-1, and -2) have the same target (mouse GAPDH mRNAs) showed a clear correlation of the knockdown efficiency of target mRNAs and MxA upregulation. siRNAs tageting mouse GAPDH mRNA and 28S rRNA also showed a dose-dependent effect of MxA induction. Thus, RNAi itself or factors produced during the RNAi process may play an important role in the RNAi-mediated induction of ISGs. Two pieces of evidence support this notion: (1) silencing of viral and host genes enhanced the expression of cellular antiviral proteins such as IFN-β, MxA, and IP-10; and (2) no ISGs were induced in the absence of targeted RNAi against a specific gene.

Because RNAi results in the cleavage and degradation of target RNAs into small pieces [1] and RNase L-generated small RNAs are able to activate IFN-β [15], it is reasonable that RNAi-directed small RNA cleavage products of various lengths may also induce IFN-β activation.

2′-O-methyl oligonucleotides complementary to siRNA can act as irreversible, stoichiometric inhibitors of small RNA function [23]. Sequence-specific 2′-O-methyl oligonucleotides were used in this study to block the function of siWHx. As expected, 2′-O-methyl oligonucleotides blocked the inhibitory effect of siWHx on WHV and the upregulation of IFN-β/ISGs (MxA, IP-10) (Fig. 2). However, siWHx-mediated IFN-β/ISGs upregulation occurred later than IFN-β/ISGs upregulation mediated by direct stimulation by dsRNA (Fig. 3). This phenomenon can be explained by the finding that it takes hours for siRNA to cleave target RNA and produce sufficient small RNA pieces to activate RNA adaptors such as PKR, TLR3, and TLR7/8 [24]. These data support the notion that the RNA cleavage products in the RNAi process contribute to IFN-β activation and the induction of ISGs. Endogenous fragments of RNA cleavage products could also initiate IFN-β production to a lesser extent, although this scenario may not occur under physiological conditions because siRNA targeting endogenous RNA is not physiologically present in mammals [25].

Studies have reported nonspecific changes in the expression of interferon-stimulated genes in response to the delivery of siRNAs [26]–[29]. Some siRNAs induce IFN-β responses in cells in a sequence-specific manner and act through TLR7/8 pathways. Immunostimulatory motifs like 5′-UGUGU-3′, 5′-GUCCUUCAA-3′, UGGC, GU, and AU on the sense strand of siRNAs were described [30]–[38]. In general, uridine/guanosine and uridine/adenosine nucleotides within immunostimulatory RNA activate TLR7/8 sensors, respectively [39]. Blunt-ended synthetic siRNAs activate the IFIT1 gene mediated by RIG-I [40]. Finaly, siRNA delivery vehicles will influence the immunostimulatory properties of siRNAs or themselves activate immune responses [39], [41]. The combination of both gene-silencing and immunostimulation in one RNA molecule may be useful as novel drugs for effective treatment of viral infection and cancer [42], [43]. On the other hand, it is showed that 24-bp fragments of perfect dsRNA and fragments with short ssRNA overhangs do not activate IFN [44]. In our studies, the siRNAs (e.g., siWHx, siGAPDH, siActin) never induced IFN-β in the absence of their target RNAs in primary hepatocytes of woodchuck [14] and mouse origin (Fig. S1), which thereby excludes the possibility of direct stimulation by the siRNAs themselves.

Malathi et al. found that RNase L-generated small RNAs activate IFN-β through the MDA5/IPS1 signaling pathway [15]. In the present study, RIG-I and IPS1 knockdown using specific siRNAs did not affect the siWHx-directed induction of ISGs (Fig. S2), which excludes the possible involvement of the MDA5/IPS1 signaling pathway. Therefore, other pathways must have been triggered in the RNAi-directed activation of IFN-β. In addition to the MDA5/IPS1 signaling pathway, at least 3 other pathways can recognize RNA molecules and induce IFN-β activation: the PKR signaling pathway and the TLR3 and TLR7/8 signaling pathways. The RNA-activated protein kinase PKR is an interferon-induced protein that is part of the innate immune response and inhibits viral replication. The action of PKR involves RNA-dependent autophosphorylation that results in the inhibition of translation. PKR has an N-terminal dsRNA-binding domain that can interact in a non-sequence-specific manner with long (>33 bp) stretches of dsRNA and thus lead to activation. In addition, certain viral and cellular RNAs containing non-Watson–Crick structures and multiple, shorter dsRNA sections can regulate PKR [45]. 2-AP is a potent inhibitor of the kinase activity of PKR at millimolar concentrations and is widely used as an inhibitor of PKR [46]. The presence of 2-AP blocked siWHx-mediated upregulation of MxA in a concentration-dependent manner (Fig. 4) and blocked RNAi-directed PKR phosphorylation (Fig. 5B). This finding could be verified with an alternative PKR inhibitor PKR-I. These results provide evidence that the PKR signaling pathway is involved in RNAi-directed IFN-β stimulation.

Chloroquine is a lysosomotropic agent that prevents endosomal acidification and is commonly used to study the role of endosomal acidification in cellular processes, such as the signaling of intracellular TLRs including TLR3, TLR7/8, and TLR9 [47], [48]. TLRs 3, 7/8 and 9 reside in the endosomal compartment and specifically recognize viral or host-derived nucleic acids. Among these TLRs, TLR3 recognizes dsRNA, which is associated with viral infection. Poly I:C is a synthetic analog of dsRNA and is the ligand of choice for TLR3. TLR7 and TLR8 are involved in the response to viral infections and recognize GU-rich short single-stranded RNA as well as small synthetic molecules such as midazoquinolines and nucleoside analogues. After ligand binding, the cytoplasmic TIR domain of TLRs associates with intracellular adaptors and activates downstream signaling molecules, including the transcription factors NF-κB, IRF1/3/7, JNK, and MAPKs, which leads to the activation of type I IFNs, pro-inflammatory cytokines, chemokines, adhesion molecules, antibodies, MHCs, or co-stimulatory molecules [49]. The pretreatment of PWHs with chloroquine at least partially blocked the RNAi-directed upregulation of MxA in a concentration-dependent manner (Fig. 4A), which suggests that TLR3 and TLR7/8 partially contribute to RNAi-directed MxA induction.

**Figure 6:**
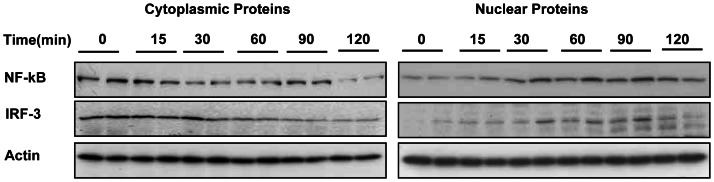
Nuclear translocation of IRF3 and NF-κB in the RNAi process. PWHs isolated from woodchucks with chronic WHV infection were transfected with 100 nM siWHx, and cytosolic and nuclear proteins were prepared at 0, 15, 30, 60, 90, and 120 minutes posttransfection. Cytosolic and nuclear IRF3 and NF-κB were detected by Western blotting.

The activation of transcription factors IRF3/5/7 and NF-κB results in the induction of type I IFNs. Upon activation, IRFs are phosphorylated, dimerized, and translocated into the nucleus, whereas NF-κB disassociates from IkB and translocates into the nucleus [50]. In the present study, treatment of PWHs with siWHx resulted in the nuclear translocation of IRF3 and NF-κB (Fig. 6), which suggests that IRF3 and NF-κB were activated during the RNAi-mediated induction of IFN-β/ISGs.

**Figure 7:**
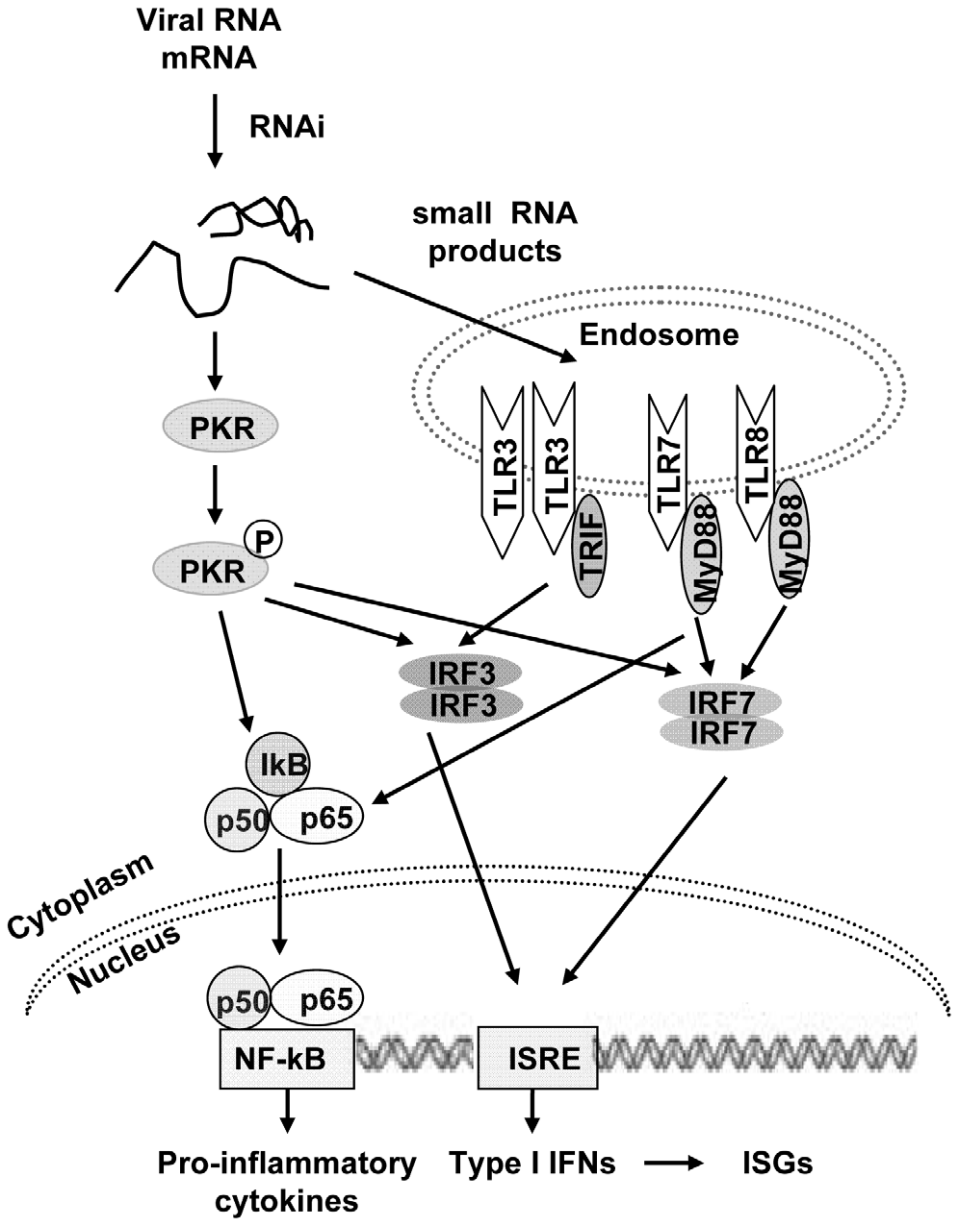
Model for transcriptional activation of the IFN-β gene by RNAi. The small RNAs generated in the RNAi process may, on the one hand, activate PKR, leading to PKR auto phosphorylation, and on the other hand, enter the endosome and recognized by TLR-3, −7/8. Both the PKR activation and TLR-3, −7/8 signaling will lead to nuclear translocation of IRF3/7 and NF−κB which, in turn, stimulate production of type I IFNs and pro-inflammatory cytokines.

Taken together, these findings show that RNAi is capable of enhancing IFN-β production through the PKR and TLR3/7/8 cascade. The RNAi-generated small viral RNA cleavage products, which consist of ‘non-self’ G-U rich RNA molecules, may signal through PKR and TLR3/7/8 to expand the antiviral RNAi (Fig. 7). RNAi amplify innate immunity and provide sequence-specific silencing of pathogens, which makes RNAi a ‘dual activity’ approach with direct antiviral and immunoenhancing activity. Thus, RNAi may serve as a potent antagonist during viral infection. RNAi-mediated IFN induction may potently enhance the anti-HBV efficiency of specific siRNAs, and further studies will focus on the in vivo effects of this dual activity approach and how to manipulate RNAi to manage viral infection while inhibiting the immunoenhancing effect of RNAi to prevent unwanted side effects.

## Supporting Information

Figure S1 **Interference targeted against housekeeping genes led to upregulation of MxA in PMHs from C57BL/6 mice.** PMHs isolated from C57BL/6 mice were transfected with 100 nM siWHx or the mouse-specific siRNAs siActin, siGAPDH, or siGFP and poly I:C as controls, and MxA RNA levels were determined by real-time RT-PCR at 48 h posttransfection.
(TIFF)

Figure S2 **The effect of RIG-I/MDA5 interference on RNAi-mediated induction of MxA.** PMHs isolated from WHV Tg mice were transfected with 100 nM siWHx or cotransfected with 100 nM mouse-specific siRNA (siRIG-I, siMDA5, or siIPS1), and the mRNA levels of mouse MxA and protein levels of mouse RIG-I were determined 48 h posttransfection by real-time RT-PCR and Western blotting, respectively.
(TIFF)
