# Supplementary figures and images for: RNAi Induces Innate Immunity through Multiple Cellular Signaling Pathways
Source: PLoS One. 2013 May 20;8(5):e64708. doi: 10.1371/journal.pone.0064708 (PMC3659100; doi:10.1371/journal.pone.0064708)

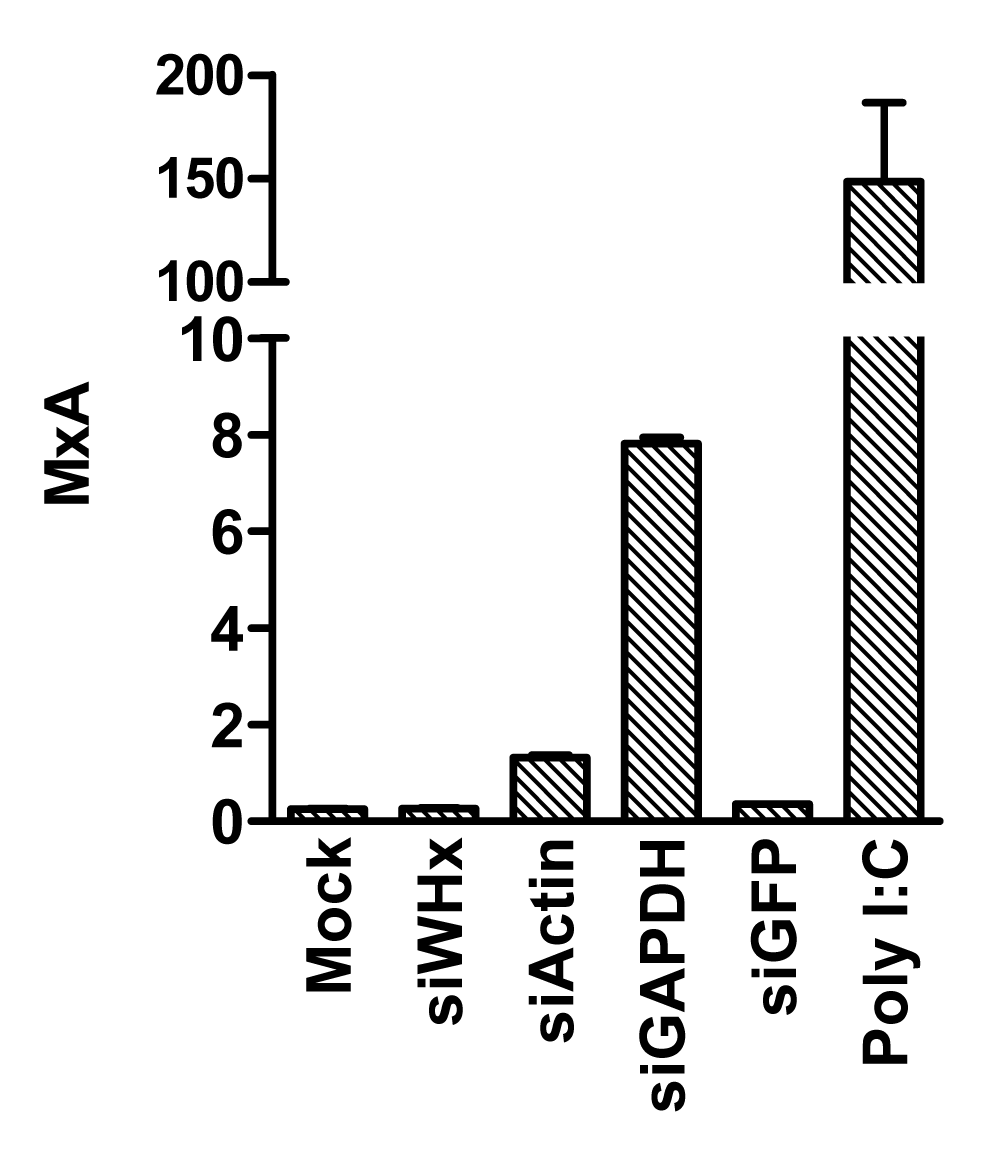

Supplement: Figure S1 — Interference targeted against housekeeping genes led to upregulation of MxA in PMHs from C57BL/6 mice. PMHs isolated from C57BL/6 mice were transfected with 100 nM siWHx or the mouse-specific siRNAs siActin, siGAPDH, or siGFP and poly I:C as controls, and MxA RNA levels were determined by real-time RT-PCR at 48 h posttransfection. (TIFF) [file pone.0064708.s001.tiff]

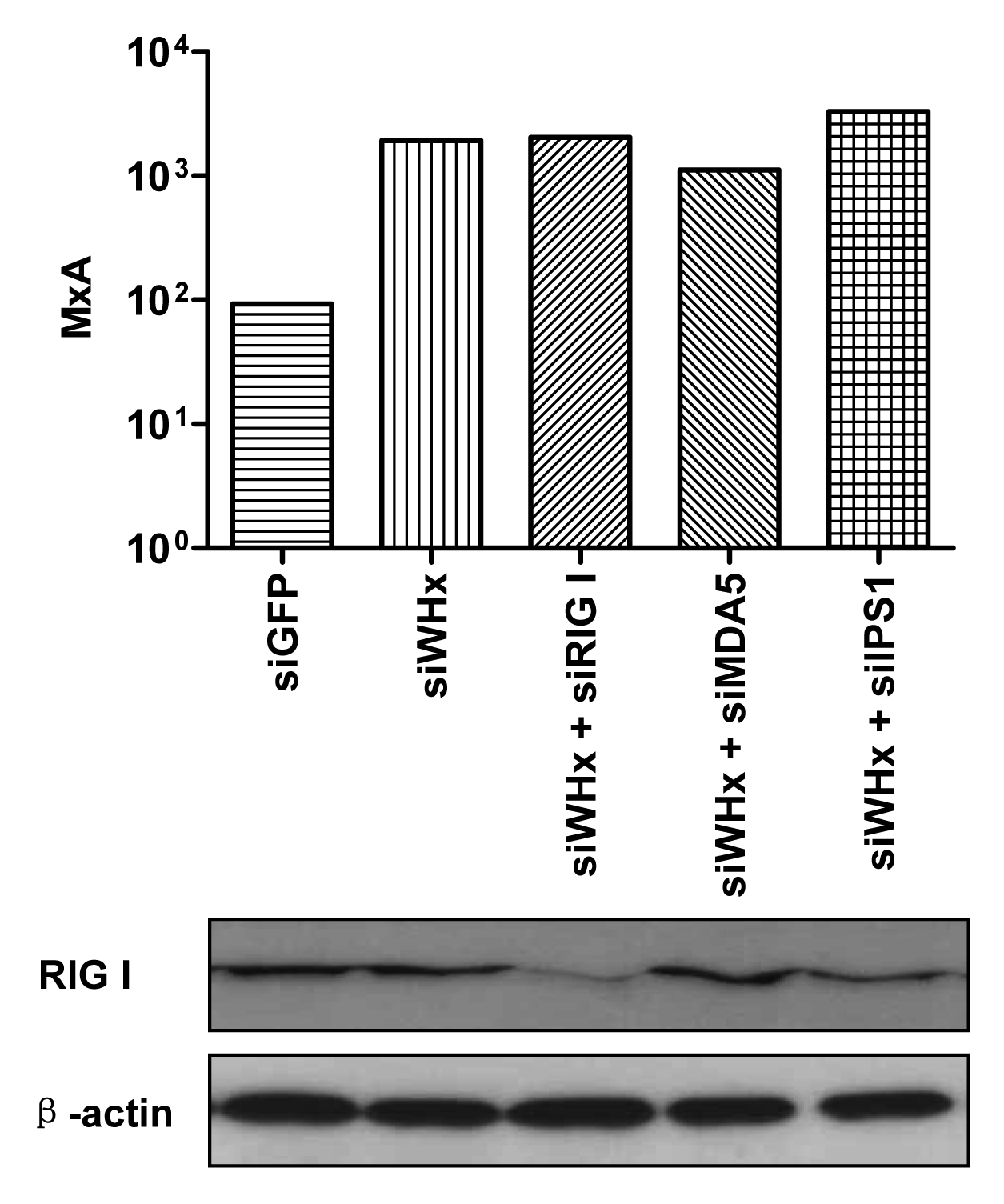

Supplement: Figure S2 — The effect of RIG-I/MDA5 interference on RNAi-mediated induction of MxA. PMHs isolated from WHV Tg mice were transfected with 100 nM siWHx or cotransfected with 100 nM mouse-specific siRNA (siRIG-I, siMDA5, or siIPS1), and the mRNA levels of mouse MxA and protein levels of mouse RIG-I were determined 48 h posttransfection by real-time RT-PCR and Western blotting, respectively. (TIFF) [file pone.0064708.s002.tiff]
